# Efect of tobacco smoking on outcomes of trabeculectomy

**DOI:** 10.5935/0004-2749.2021-0061

**Published:** 2022-09-06

**Authors:** Nadia Rios Acosta, Shveta Bali, Jennifer Rahman, Gdih Gdih, Lisa Gould

**Affiliations:** 1 Hospital San Jose de Queretaro. Santiago de Queretaro, Queretaro, Mexico; 2 University of Ottawa, Ottawa, Ontario, Canada; 3 Department of Ophthalmology, Max Rady College of Medicine, University of Manitoba, Winnipeg, Manitoba, Canada

**Keywords:** Glaucoma open-angle, Trabeculectomy, Intraocular pressure, Tobacco use disorder, Tobacco/adverse effects, Visual acuity, Glaucoma de ângulo aberto, Trabeculectomia, Pressão intraocular, Tabagismo, Tabaco/efeitos adversos, Acuidade visual

## Abstract

**Purpose:**

To evaluate the effect of tobacco smoking on trabeculectomy outcomes.

**Methods:**

Charts of patients with glaucoma who underwent trabeculectomy performed by a single surgeon between 2007 and 2016 were retrospectively reviewed. Charts were screened for a documented history of smoking status before surgery. Demographic and clinical preoperative variables were recorded. Based on smoking history, subjects were divided into two groups: smokers and nonsmokers. Any bleb-related interventions (e.g., 5-flourouracil injections ± laser suture lysis) or bleb revision performed during the postoperative period were noted. Success was defined as an intraocular pressure >5 mmHg and <21 mm Hg without (complete success) or with (qualified success) the use of ocular hypotensive medications. Failure was identified as a violation of the criteria mentioned above.

**Results:**

A total of 98 eyes from 83 subjects were included. The mean age of the subjects was 70.7 ± 11.09 years, and 53% (44/83) were female. The most common diagnosis was primary open-angle glaucoma in 47 cases (47.9%). The smokers Group included 30 eyes from 30 subjects. When compared with nonsmokers, smokers had a significantly worse preoperative best-corrected visual acuity (p=0.038), greater central corneal thickness (p=0.047), and higher preoperative intraocular pressure (p=0.011). The success rate of trabeculectomy surgery at 1 year was 56.7% in the smokers Group compared with 79.4% in the Group nonsmokers (p=0.020). Smoking presented an odds ratio for failure of 2.95 (95% confidence interval, 1.6-7.84).

**Conclusion:**

Smokers demonstrated a significantly lower success rate 1 year after trabeculectomy compared with nonsmokers and a higher requirement for bleb-related interventions.

## Abstract (pt)

**Objetivo:**

Avaliar o efeito do tabagismo nos desfechos da trabeculectomia.

**Métodos:**

Uma revisão retrospectiva do gráfico de pacientes com glaucoma submetidos à trabeculectomia foi realizada por um único cirurgião entre 2007 e 2016. Os gráficos foram examinados para uma história documentada de condição de fumante antes da cirurgia. Variáveis pré-operatórias clínicas e demográficas e clínicas foram registradas. Os pacientes foram divididos em dois grupos de acordo com sua história de tabagismo em fumantes e não fumantes. Quaisquer Intervenções relacionadas à bolha, por exemplo, injeções de 5-fluorouracil + lise de sutura com laser, ou revisão da bolha realizada durante o período pós-operatório foram observadas. O sucesso foi definido como pressão intraocular > 5 mmHg e < 21 mm Hg sem (sucesso completo) ou com (sucesso qualificado) medicamentos hipotensores oculares. A falha foi identificada como violação dos critérios mencionados acima.

**Resultados:**

O estudo incluiu 98 olhos de 83 pacientes com idade média de 70,7 ± 11,09 anos, sendo 53% (44/83) dos pacientes do sexo feminino. O diagnóstico mais comum foi o glaucoma de ângulo aberto primário com 47 casos (47,9%). O Grupo de fumantes incluiu 30 olhos de 30 pacientes. Os fumantes, quando comparados aos não fumantes, apresentaram uma melhor acuidade visual pré-operatória significativamente pior (p=0,038), maior espessura central da córnea (p=0,047) e maior pressão intraocular pré-operatória (p=0,011). A taxa de sucesso de um ano para a cirurgia de trabeculectomia foi de 56,7% no Grupo de fumantes contra 79,4% no Grupo de não fumantes (p=0,020). O tabagismo apresentou razão de chances para falha de 2,95 95% de IC (1,6-7,84).

**Conclusão:**

Os fumantes demonstraram uma taxa de sucesso significativamente menor em um ano após a trabeculectomia em comparação com os não fumantes e uma maior necessidade de intervenções relacionadas à bolha.

## INTRODUCTION

Glaucoma is the second leading cause of blindness worldwide and the most common cause of irreversible blindness^(1)^. With the aging of the global population, glaucoma is expected to become increasingly important in the years to come.

Intraocular pressure (IOP) is currently the only modifiable risk factor for the progression of glaucoma. IOP is treated through pharmacologic therapy, laser procedures, and, when all conservative options for treatment have been exhausted, filtering surgery, which is the gold standard for uncontrolled glaucoma.

The most common initial surgical technique for treating refractory glaucoma is trabeculectomy. This procedure consists of the creation of an ostium beneath a partial-thickness scleral flap to bypass the dysfunctional trabecular meshwork, creating an alternative pathway into the subconjunctival space, which follows a trans-venous or transconjunctival route for further drainage into the bloodstream.

Trabeculectomy success depends on a delicate balance between healing and antihealing mechanisms. Although inadequate healing may lead to complications such as wound leak, overfiltration, and/or hypotony, the most common cause of failure is an excessive healing response that results in scarring under the scleral flap or conjunctiva-Tenon’s capsule complex^(2)^.

Pharmacologic agents have become frequently used to modulate this healing response. Topical steroids and antimetabolites such as mitoMYcin C and 5-fluorouracil (5-FU) are particularly used.

There are several risk factors for trabeculectomy failure due to scarring, including secondary glaucoma, previous conjunctival surgery, long-term use of a preservative containing topical glaucoma therapy, age less than 40 years, African-Caribbean descent, diabetes, previous ocular inflammation, and anterior segment neovascularization^(3)^.

Tobacco smoke is a known health hazard and a source of more than 4000 toxic chemical substances^(4)^. It has been systematically associated with cancer, cardiovascular disease, and chronic obstructive pulmonary disease, among many other life-threatening conditions. In ophthalmology, it has been associated as a risk factor for dry eye disease^(5)^, age-related macular degeneration^(6)^, intraocular inflammation^(7)^, cataract^(8)^, glaucoma^(9)^, thyroid eye disease, anterior ischemic optic neuropathy and, retinal vein occlusion^(10)^.

The Collaborative Initial Glaucoma Treatment Study group found that smokers who underwent surgery had a higher mean IOP over time than nonsmokers did^(11)^. Although smoking affects both the extraocular and intraocular milieu of the eye, there have been no published studies to date regarding the effect this might have on trabeculectomy success.

## METHODS

The study protocol was reviewed and approved by the Institutional Review Board of Bannatyne Campus Research Ethics Board at the University of Manitoba (Winnipeg, MB, Canada). The study followed the tenets of the Declaration of Helsinki.

We performed a retrospective chart review of all subjects who underwent simple trabeculectomy with the application of mitomycin C performed by a single surgeon from 2007 to 2016.

We reviewed the charts of subjects undergoing the procedure as a first filtrating surgery and included the patients’ clinical history of tobacco smoking. Incomplete clinical files and charts with no information on smoking status were excluded, as were cases of secondary glaucoma. Patients who quit smoking any time before or during the postoperative period and those with previous conjunctival surgery were also excluded.

We collected the following preoperative data from the chart: demographics, preoperative diagnosis, central corneal thickness (CCT), and number of smoking pack-years (PY), presurgery IOP by Goldmann applana-tion tonometry, duration of glaucoma diagnosis before surgery, number of ocular hypotensive medications, cup-to-disc ratio (CDR), and best-corrected visual acuity (BCVA) on the LogMAR scale. Postoperative variables, including IOP, complications, and bleb-related interventions (namely, 5-FU injections or suture lysis), were collected from day 1, week 6, month 6, and year 1 of follow-up.

Surgical success was defined as an IOP >5 mmHg and <21 mmHg without (complete success) or with (qualified success) the use of ocular hypotensive medications. Failure was identified as violation of the above criteria.

For the analysis, we divided the subjects into two groups based on their smoking history: smokers and nonsmokers. A subject was considered a smoker if he or she had at least a 1-year history of tobacco smoking and smoked at least one cigarette, cigar, or pipe per day.

### Surgical technique

All subjects underwent fornix-based trabeculectomy with a consistent technique as follows. Xylocaine 2% nonpreserved gel was applied to the ocular surface before prepping and draping. A superior corneal 6-0 Vicryl traction suture was used to position the eye and rotate it downward. At the limbus, at the 3-o’clock position temporal to the surgical site, 2% on-preserved lidocaine was infiltrated with a 30-g needle into the subconjunctival space. To spread the anesthetic into the nasal quadrant, a cotton swab was used to massage this area along the limbus. Dull Westcott scissors and nontoothed forceps were then used to create a fornix-based limbal incision through the conjunctiva and Tenon’s at the 4.5-o’clock position in length. The area was dissected into the superonasal quadrant, and additional nonpreserved 2% lidocaine was then instilled using a blunt cannula. Bleeding episcleral vessels were scraped off with a 69-feather blade, and the remaining vessels were cauterized. A 4- x 4-mm, half-scleral-thickness triangular flap was created until clear cornea was reached. Two corneal shields soaked in 0.2 mg/ml mitomycin C were placed under and posterior to the flap for 2 minutes. Subsequently, the area was rinsed thoroughly with 40 ml of balanced saline solution. Using a feather blade, an ostium (0.2 x 0.2 mm in size) was created, and an iridectomy was performed. Depending on the apposition of the tissues, the scleral flap was sutured with one to three 10-0 Nylon sutures. The conjunctiva was closed using two corneal winged sutures with 10-0 Nylon. Any radial extension of the conjunctival incision was further closed with interrupted 10-0 Nylon sutures, as required.

### Statistical analysis

We performed all statistical analyses using IBM SPSS Statistics for Windows, version 19 (IBM Corp., Armonk, NY, USA). Continuous variables were compared between the two groups using a one-way analysis of variance. We used the chi-square test and Fisher’s exact test to compare binomial variables. A generalized estimating equation multivariate analysis model was used to account for repeated measures within subjects. One-year survival was plotted into a Kaplan-Meier curve.

## RESULTS

The study included 98 eyes from 83 subjects. The mean age of the subjects was 70.7 ± 11.09 years, and 53% (44/83) were female (Table 1). The most common diagnosis was primary open-angle glaucoma in 47 cases (47.9%), followed by pseudoexfoliative glaucoma in 35 cases (35.7%; Table 2).

**Table 1:** Comparison of baseline preoperative characteristics between the two groups.^a^

	Smokers n=30	Nonsmokers n=68	p-value
	Mean ± SD	Mean ± SD	
Age	69.4 ± 9.2	71.3 ± 11.8	0.443
Gender, % female (n)	43.3% (13)	57.4% (39)	0.144
Presurgery IOP (mmHg)	28.4 ± 9.0	23.3 ± 8.8	0.011
History of DM	13.3% (4)	14.7% (10)	0.57
Number of glaucoma drops before surgery	3.4 ± 0.77	3.5 ± 0.98	0.491
Length of diagnosis (years)	8.3 ± 4.5	9.0 ± 6.6	0.614
CCT (µm)*	551.7 ± 40.5	535.41 ± 35.3	0.047
Cup-disc ratio	0.83 ± 0.21	0.87 ± 0.16	0.367
Presurgery BCVA (LogMAR)	0.40 ±.0.3	0.26 ± 0.3	0.038

DM= diabetes mellitus; BCVA= best-corrected visual acuity; CCT= central corneal thickness.

a = There were no significant differences between the groups, with the exception of CCT (p=0.047).

**Table 2:** Distribution of Preoperative Diagnoses.^a^

Preoperative diagnosis frequency, % (n)	Smokers n=30	Nonsmokers n=68	p- value
	% (n)	% (n)	
Primary open-angle glaucoma	40% (12)	51.5% (35)	0.326
Pseudoexfoliative glaucoma	40% (12)	33.8% (23)	
Mixed mechanism glaucoma	6.7% (2)	7.4% (5)	
Pigmentary dispersion glaucoma	6.7% (2)	1.5% (1)	
Chronic angle closure glaucoma	0	4.4% (3)	
Pseudophakic glaucoma	6.7% (2)	1.5% (1)	

a Most common diagnoses were primary open-angle glaucoma and pseudoexfoliative glaucoma for both groups.

A total of 30 eyes were from 30 subjects (30.6%) classified as smokers. There were no statistically significant differences in age, gender, number of glaucoma drops, length of diagnosis, or CDR between the smoker and nonsmoker Groups.

When compared with nonsmokers, smokers had a significantly worse preoperative BCVA (0.40 ± 0.3 vs 0.26 ± 0.3, p=0.038), a higher CCT (551.7 ± 40.5 µm vs 535.41 ± 35.3 µm, p=0.047), and higher preoperative IOP (28.4 ± 9.0 mmHg vs 23.3 ± 8.8 mmHg, p=0.011; Table 1). Data on PY were reported in 17 of 30 charts (26.4 ± 22.41 PY; median 10 [range, 6-57 PY]). Because of the small sample and wide variability, we excluded the latter variable from the final analysis.

Within the first year after trabeculectomy surgery, bleb-related interventions were required in 33.3% (10/30) of the eyes in the smoker group compared with 14.7% (10/68) of the eyes in the nonsmoker Group (p=0.036; Table 3). Of the cases requiring intervention, three underwent laser suture lysis and all underwent 5-FU injections.

**Table 3:** End-line results for both groups.^a^

	Smokers n=30	Nonsmokers n=68	p- value
One-year success	56.7% (17)	79.4% (54)	0.020
Overall BRI	33.3% (10)	14.7% (10)	0.036
Successful cases among BRI	50% (5)	70% (7)	0.015
IOP at last follow-up, mean ± SD	18.2 ± 8.5	15.0 ± 6.3	0.040
Number of drops at last follow-up, mean ± SD	1.2 ± 1.2	0.92 ± 1.3	0.34

BRI= bleb-related interventions.

a The 1-year success rate was significantly higher in the nonsmoker group (p=0.020). There was a significantly higher frequency of bleb-related interventions among smokers (p=0.036), with higher rates of success among nonsmokers (p=0.015). After 1 year, the IOP was significantly higher in the smoker group (p=0.040).

**Figure 1:**
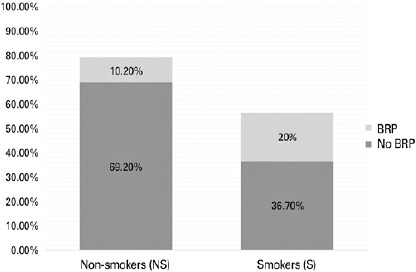
Kaplan–Meier curve of the 1-year success rate of both study groups. The nonsmoker group showed a significantly higher survival rate for trabeculectomy at 1-year follow-up. Mantel–Cox analysis, p=0.015.

The one1year success rate for trabeculectomy surgery was 56.7% (17/30 eyes) in the smoker group compared with 79.4% (54/68 eyes) in the nonsmoker group (p=0.015; Figure 1). Smoking presented an odds ratio for failure of 2.95 (95% confidence interval [CI], 1.6-7.84]). Multivariate analysis showed that preoperative IOP was correlated with the likelihood of surgical success (p=0.021).

**Figure 2:**
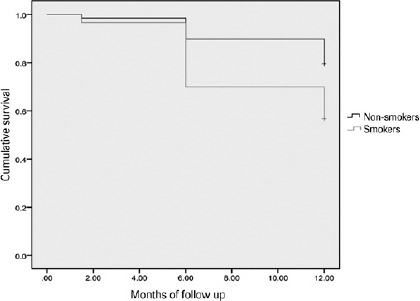
One-year trabeculectomy survival among the study groups.

Bleb-related interventions aided in the survival of 35.2% (6/17) and 12.9% (7/54) of the successful cases in the smoker and nonsmoker Groups, respectively (Figure 2). In addition, the cases requiring these procedures had an average last IOP measurement 5 mmHg higher (95% CI 1.5-8.0 mmHg) than those who did not receive an intervention (p=0.002) as well as a higher number of topical medications needed at the time of last follow-up (p=0.001).

Percentage of success attributable to BRP (Bleb-related Procedures) displayed in red. NS Group presented an overall 79.4% of success compared to the S Group 56.7%.

NS: NonsmokersS: Smokers.

## DISCUSSION

The current study analyzed the effect of tobacco smoking on the success of trabeculectomy. When compared with the nonsmokers, the success of smokers was significantly lower, and these subjects required more bleb-related interventions to aid in the survival of the surgery.

Subjects who smoked had a significantly higher preoperative IOP, which in turn correlated with a lower possibility of success. A previous study described a higher average IOP in association with tobacco smoking in healthy subjects and patients with glaucoma^(12)^. The physiopathogenic basis behind this finding has been suggested to be related to the effect of smoking on corneal hysteresis^(13)^, choroidal thickness^(14)^, and an increase in episcleral venous pressure resulting from vasoconstric-tion. Zanon-Moreno et al.^(15)^ suggested that the liberation of free radicals due to smoking causes damage in the trabecular meshwork, decreasing the outflow of the aqueous humor. In our study, this finding-along with the lack of preoperative difference in age, length of diagnosis, CDR, and number of glaucoma drops between both groups-may suggest a more aggressive IOP in patients who have a poorer response to medical treatment. Although the numbers are too small to draw conclusions, the higher preoperative IOP without a corresponding larger CDR raises the question of a possible protective effect in smokers. The United Kingdom Glaucoma Treatment Study found that smokers had better visual field preservation, with a possible reason suggested as a neuroprotective effect of tobacco smoke^(16)^. In addition, a protective effect of tobacco smoke has been postulated in other chronic neurologic diseases, such as Parkinson’s^(17)^. However, because of the mixed and complex findings, more research is required regarding the possible association between tobacco smoke and neuroprotection in glaucoma.

Smokers also showed a higher CCT. Although Wang et al. reported that smoking could be associated with lower CCT^(18)^, this claim was not supported by the findings of our study or by subsequently published literature^(19)^. It could be argued that this parameter influenced IOP, because a higher CCT often results in a higher IOP reading in applanation tonometry; however, the magnitude of the difference in IOP was too high to be explained by this factor alone.

Interestingly, despite the similar duration of diagnosis before surgery and CDR, the smoker group also had a significantly lower mean BCVA. Because of the design and scope of our study, we cannot make a valid assumption as to the impact of tobacco smoking on the affectation of the central visual field due to glaucoma; however, smoking has been found to have a negative effect on visual acuity in patients with conditions such as retinitis pigmentosa and age-related macular degeneration, in which a possible cause has been postulated to be the chronic inflammation and oxidative stress resulting in cone cell death^(20)^.

Among smokers, the overall survival rate at 1 year of followe-up was significantly lower than the rest of the cohort, and these subjects presented almost three times the risk for failure in comparison (odds ratio 2.95; 95% CI, 1.6-7.84). Accordingly, bleb-related interventions were more frequently required and salvaged almost one-third of the successful cases in the smoker Group. We propose three possible mechanisms to explain these findings: an increased inflammatory response, histological changes in the conjunctiva, and local microvascular alterations.

The increased need for bleb-related interventions could be related to the proinflammatory effect of tobacco smoking. Tobacco smoking increases inflammatory markers, such as interleukin-6 (IL-6) in several tissues and fluids, including tear film and aqueous humor^(21)^. Patients with glaucoma already demonstrate elevated cytokines in aqueous humor, and this has been found to be exacerbated in patients with a history of active smoking^(15)^. Because higher preoperative levels of tumor necrosis factor-α and IL-6 in aqueous humor are associated with worse outcomes of glaucoma surgery^(22)^, the induction of extraocular and intraocular inflammation could result in a higher scarring response among patients who smoke tobacco.

In functional trabeculectomy, the histological integrity of the conjunctiva is important. Aqueous microcysts, which possibly correspond to goblet cells filled with aqueous humor^(23)^, correlate with successful functioning blebs with a lower postoperative IOP^(24)^. The density of goblet cells has been associated with the success of filtration surgery, both preoperatively and postoperative-ly^(25^,^26)^. Cigarette smoking, which causes a significant loss of goblet cells, neutrophilic infiltration, and squamous metaplasia, has a proven deleterious effect on the ocular surface and tear film and a targeted effect on the histology of the conjunctiva^(27)^. These alterations have a possible synergistic effect, adding up to less functional blebs with a lower time of survival than that of nonsmokers.

Finally, the outflow of the aqueous humor through the trabeculectomy and filtering bleb requires a functional vessel network to be drained into the systemic circulation. Previous researchers indicated that tobacco smoking alters the microcirculation of the conjunctiva, causing endothelial dysfunction^(28)^ as well as affecting the episcleral venous pressure increase due to vasocons-triction^(13^,^14)^. Another factor in the failure of these cases might be the restriction of the outflow of aqueous humor beyond the trabeculectomy site.

Smoking has been widely correlated with the glaucoma development^(9^,^29)^. Our findings demonstrate that smokers are likely to present with a more aggressive IOP requiring filtrating surgery, which at the same time will require a closer follow-up and possibly earlier and more frequent interventions to improve the survival of the trabeculectomy. Another possible option for these patients that should be investigated is the use of drainage implants; however, there are reports that smoking is a risk factor for drainage implant erosion^(30)^. Hence, regardless of the surgical option, the care of these patients will likely result in an increased number of office visits, along with possibly more expensive care and a path of multiple interventions throughout the patient’s life.

To our knowledge, this is the first study to evaluate the effect of smoking on trabeculectomy outcomes. The main strength of our study is that the procedures and clinical care were performed based on the criteria of a single surgeon, which provides a fair uniformity to this process. However, because of the retrospective nature of the study, our research has a few limitations that should be acknowledged. First, the sample size of the smokers was too low to provide an ideal comparison. Second, the smoking exposure could not be quantified in terms of PY in all subjects and the variability was high for the sample size. Finally, we were unable to evaluate the effect of passive smoking. Further prospective, histological, and physiological studies are required to elucidate whether these findings are reproducible and to confirm the possible causes.

This study suggests that, as compared with not smoking, smoking is associated with a significantly lower 1-year success rate after trabeculectomy and an increased need for postoperative procedures such as 5-FU injections and suture lysis for surgical success. The possible mechanisms underlying these findings include the proinflammatory effect of tobacco smoke, its deleterious effects in the conjunctival histology, and its vascular effects in the regional microvasculature. To further evaluate the effect of this potentially significant risk factor on outcomes of glaucoma filtration surgery, future prospective studies with larger size samples are required.
